# Individual differences in skewed financial risk-taking across the adult life span

**DOI:** 10.3758/s13415-017-0545-5

**Published:** 2017-10-23

**Authors:** Kendra L. Seaman, Josiah K. Leong, Charlene C. Wu, Brian Knutson, Gregory R. Samanez-Larkin

**Affiliations:** 1 Center for Cognitive Neuroscience, Duke University, Durham, NC 27708 USA; 2 Department of Psychology, Stanford University, Palo Alto, CA 94305 USA; 3 Department of Psychology and Neuroscience, Duke University, Durham, NC 27708 USA; 4 Uber Labs, San Francisco, CA USA

**Keywords:** Aging, Decision-making, Skew, Fraud, Neuroimaging

## Abstract

Older adults are disproportionately targeted by fraud schemes that advertise unlikely but large returns (positively skewed risks). We examined adult age differences in choice and neural activity as individuals considered risky gambles. Gambles were symmetric (50% chance of modest win or loss), positively skewed (25% chance of large gain), or negatively skewed (25% chance of large loss). The willingness to accept positively skewed relative to symmetric gambles increased with age, and this effect replicated in an independent behavioral study. Whole-brain functional magnetic resonance imaging analyses comparing positively (vs. negatively) skewed trials revealed that relative to younger adults, older adults showed increased anticipatory activity for negatively skewed gambles but reduced activity for positively skewed gambles in the anterior cingulate and lateral prefrontal regions. Individuals who were more biased toward positively skewed gambles showed increased activity in a network of regions including the nucleus accumbens. These results reveal age biases toward positively skewed gambles and age differences in corticostriatal regions during skewed risk-taking, and have implications for identifying financial decision biases across adulthood.

**Electronic supplementary material:**

The online version of this article (10.3758/s13415-017-0545-5) contains supplementary material, which is available to authorized users.

Given an uncertain future, nearly all long-term financial choices involve some degree of risk. Although research on financial risk-taking has explored the effects of expected value (mean) and risk (variance) on financial choice (e.g., Knutson & Huettel, 2015), less research has focused on how extreme or skewed outcomes influence choice, and no prior research has focused how aging might influence preferences for skewed risks.

Using neuroimaging tools like functional magnetic resonance imaging (fMRI), researchers have begun to investigate how skewed gambles influence affective responses and brain activity, which modulate choice (Wu, Bossaerts, & Knutson, 2011). These findings suggest that positively skewed gambles (i.e., low probability of a large gain) elicit positive arousal and NAcc activity, and are generally preferred relative to symmetric gambles, even given similar expected value and variance. Negatively skewed gambles (low probability of a large loss), however, elicit negative arousal and are sometimes preferred less than positively skewed gambles. Critically, both anticipatory NAcc activity and self-reported positive arousal have also been associated with individual differences in positive skew preference (Wu et al., 2011), which may be moderated by cortical inputs from regions like the anterior insula (AIns; Leong, Pestilli, Wu, Samanez-Larkin, & Knutson, 2016). Existing research has focused primarily on young adults’ choices, but no research has specifically focused on age differences in preferences for skewed risks. This is important because older adults are often targeted by fraudulent investment schemes (Pak & Shadel, 2011), which often promise positively skewed returns (in the form of large but unlikely financial gains). However, very little empirical research has examined age differences in fraud susceptibility (Ross, Grossmann, & Schryer, 2014), so the mechanisms underlying potential vulnerability in old age are unclear.

While a growing literature has explored adult age differences in risky choice overall (i.e., studies that consider mean and variance of gambles), it has produced mixed results (Best & Charness, 2015; Mata, Josef, Samanez-Larkin, & Hertwig, 2011). While some studies suggest that older adults accept less risk than younger adults do (e.g., Rolison, Hanoch, & Wood, 2012), others suggest the opposite (e.g., Zamarian, Sinz, Bonatti, Gamboz, & Delazer, 2008), and still others report no age differences (e.g., Wood, Busemeyer, Koling, Cox, & Davis, 2005). Some of these discrepancies may result from differing cognitive demands across tasks (Mata et al., 2011), but tasks may also vary with respect to the probability distribution of possible outcomes. Specifically, if affect drives skew preference (Wu et al., 2011), then older adults may be more likely to accept positively skewed risks (with large potential gains) based on the increasing salience of positive relative to negative incentives over the adult life span (Carstensen & Mikels, 2005; Mather & Carstensen, 2005). Further, older adults may be more likely to avoid negatively skewed risks (with large potential losses) due to a regulatory focus on maintaining and avoiding the loss of current resources (Baltes & Baltes, 1990; Freund & Ebner, 2005; Heckhausen, 1997). Neuroimaging research also suggests that incentive processing in the brain may change with age (Samanez-Larkin & Knutson, 2015). For example, while gain anticipation elicits comparable NAcc activity across adulthood, AIns activity is reduced with age during loss anticipation (Samanez-Larkin et al., 2007; Samanez-Larkin, Worthy, Mata, McClure, & Knutson, 2014). This leads us to predict that neural response in anticipation of positively skewed gambles (with large potential gains) will be preserved with age, while neural response in anticipation of negatively skewed gambles (with large potential losses) will be diminished with age. Collectively, these findings led to us to predict that age might influence both behavioral and neural responses to positively skewed and negatively skewed gambles.

These studies thus examined adult age differences in choice (Studies 1 and 2) and neural activity (Study 1) before individuals chose to accept or reject symmetric, positively skewed, or negatively skewed gambles as they underwent fMRI scanning. Our first aim was to determine whether age influenced the choice of risky skewed gambles. Based on an anticipatory affect account of financial risk-taking (Knutson, Katovich, & Suri, 2014; Wu, Sacchet, & Knutson, 2012) and on valence biases in affective processing with age (Baltes & Baltes, 1990; Mather & Carstensen, 2005), we predicted that compared to younger adults, older adults would be both more likely to accept positively skewed gambles than symmetric gambles and less likely to accept negatively skewed gambles than symmetric gambles. Our second aim was to determine if individual differences in neural activity were related to risky choice. Based on previous research in young adults, we predicted that NAcc activity would predict acceptance of gambles in general (Wu et al., 2011; Wu et al., 2012), and that this relationship would be preserved across adulthood (Samanez-Larkin et al., 2007; Samanez-Larkin et al., 2014). We also predicted that AIns activity would instead predict rejection of gambles (Knutson & Greer, 2008; Knutson et al., 2014), and that this predictive relationship would diminish with age (Castle et al., 2012; Harlé & Sanfey, 2012; Samanez-Larkin et al., 2007).

## Method

### Study 1

#### Participants

Thirty-seven healthy adults (age: *M* = 47.9 years, range: 18–85 years) were recruited from the San Francisco Bay Area community to complete a study at the Stanford Center for Cognitive and Neurobiological Imaging. Five were excluded from analyses because of head motion >2 mm during the fMRI scan, leaving a total sample of 32. Our a priori sample size calculation suggested that we needed to collect data from at least 27 subjects in order to have a power level of at least .8, to observe an effect at least as large as the linear effect of age on brain activity (*r* = .49, *f* ^2^ = .316) reported in the most similar published study of financial risk-taking across adulthood (Samanez-Larkin, Kuhnen, Yoo, & Knutson, 2010). A subset of these individuals’ behavioral and fMRI data were included in another publication that did not focus on age differences (Leong et al., 2016). Prior to participation, informed consent was obtained, and then participants completed a battery of neuropsychological, personality, and self-report measures (see Table 1). Following these tests (but often on a second day), participants underwent the neuroimaging session described below. All participants were compensated with a flat rate of $20 per hour, as well as the total amount earned on the gambling tasks. The Stanford Medical School Institutional Review Board approved all procedures.

**Table 1:** Participant characteristics

Variable	Mean (*SD*)	*r* [95% CI] with age
Sex	13 M/19 F	
Numeracy Inventory—9 items	7.78 (1.58)	−.23 [−0.54, 0.14]
Trail Making Test (B–A)	28.55 (11.35)	.28 [−0.09, 0.58]
WAIS-III Digit Span Test (forward & backward)	18 (4.44)	**−.43 [−0.68, −0.09]**
Letter–Number sequencing	11.41 (2.96)	**−.62 [−0.8, −0.34]**
Shipley Vocabulary	33.22 (4.46)	.23 [−0.13, 0.54]
*N*	32	

*Note.* Significant associations with age highlighted in bold

#### Task

**Fig. 1:**
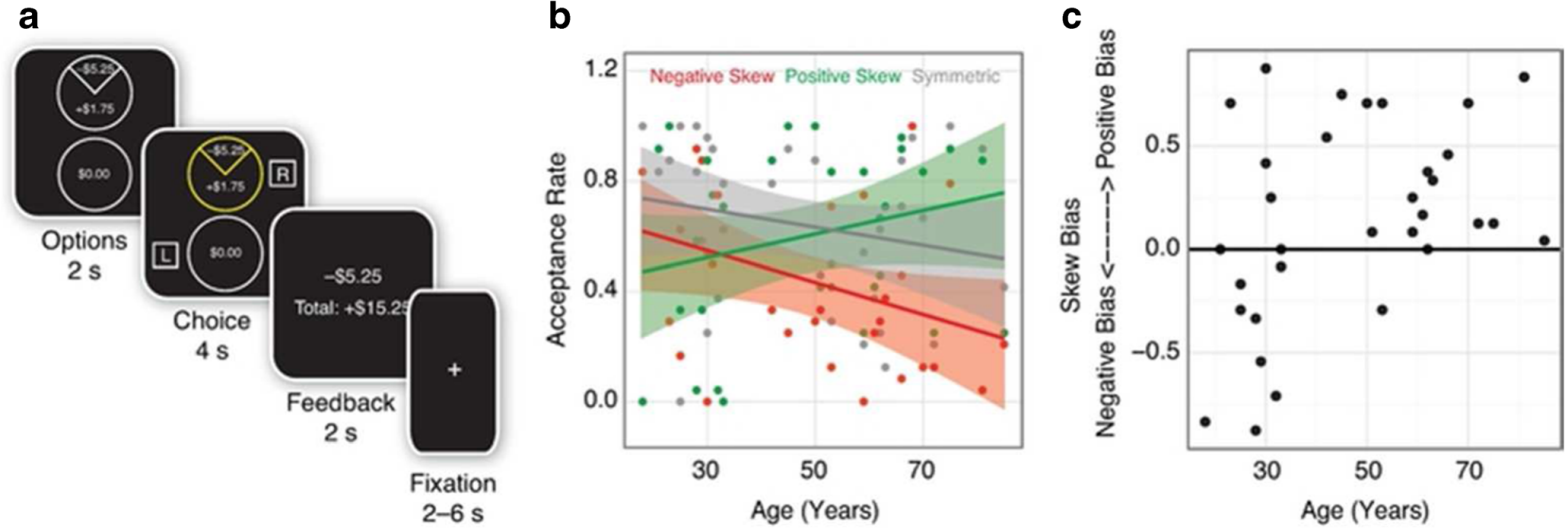
Skewed gambling task trial structure and age differences. **a** Trial structure for the gambling task. **b** Proportion of trials where the gamble was accepted over age by gamble type. **c** Difference between proportion of positively skewed and negatively skewed accepted gambles (skew bias score) over age. (Color figure online)

During fMRI acquisition, participants completed a variant of the skewed gambling task used by Wu et al. (2011). On each of 72 trials, participants viewed a gamble versus no gamble (2 s), selected one of these two options, viewed the chosen option (4 s), and then received feedback (2 s; see Fig. 1a). Participants were given feedback because it (1) is more engaging and realistic, (2) increases incentive compatibility while they are receiving money for performance on each trial, and (3) has been used and validated in many other neuroimaging tasks (O’Doherty, Cockburn, & Pauli, 2017). Trials were separated by intertrial intervals ranging from 2 to 6 seconds. Both the spatial location of the gamble (top or bottom circle) and the response associated with each circle (left or right) were counterbalanced across trials. Prior to beginning the task, participants were reminded that their choices would be played for real money and that the outcome of the gambles would determine their final payment. They were also encouraged to respond in a timely fashion so that they did not miss out on the opportunity to make money. Because explicitly stated task goals can influence responses (Maddox, Gorlick, & Worthy, 2015; Reed & Carstensen, 2012), participants were not explicitly told to try to maximize gains or avoid losses.

Three types of gambles were included: symmetric, positively skewed, and negatively skewed. Symmetric gambles featured an equal probability (50%) of winning or losing a moderate amount of money ($3.05). Positively skewed gambles featured a low probability (25%) of winning a large amount ($5.25) paired with a high probability (75%) of losing a small amount ($1.75). Negatively skewed gambles were the opposite, and featured a low probability (25%) of losing a large amount ($5.25) paired with a high probability of gaining a small amount ($1.75). Critically, the expected value of each gamble was set to $0, making it equivalent to both the alternative conditions and to a “no gamble” option, which was to accept $0 for certain, and variance was also equated across all gamble types (σ^2^ = 9.19). Participants completed 24 trials of each gamble type, presented in pseudorandom order. In other words, the three gambles described above were repeated 24 times in a mixed order, for a total of 72 trials.

Prior to the task, participants were endowed with $10 cash. On each trial, if the gamble was chosen, the gamble was played out and the results were added to the cumulative total. During the feedback period, the outcome of the gamble, or $0 if no gamble was chosen, was displayed on-screen along with the participant’s cumulative total. The cumulative total at the end of the experiment was added to or subtracted from this endowment plus the hourly rate of compensation.

#### fMRI acquisition and preprocessing

Brain images were acquired with a 3T GE Discovery MR750 scanner using a thirty-two-channel head coil. Forty-six 2.9-mm thick slices (in-plane resolution 2.9 × 2.9 mm) extending from the mid-pons to the top of the skull were acquired with an axial interleaved scheme. Functional scans were acquired using a T2*-weighted gradient pulse sequence (repetition time = 2 s, echo time = 24 ms, flip angle = 77 degrees). Anatomical scans, which were used for localization and coregistration of functional data, were acquired using T1-weighted spoiled grass sequence (repetition time = 7.2 ms, echo time = 2.3 ms, flip angle = 12 degrees, 0.9-mm isotropic voxels).

Analysis of functional neuroimaging data was conducted using Analysis of Functional Neuroimages (AFNI) software (Cox, 1996). Preprocessing of functional time series data included slice-time correction to account for nonsimultaneous acquisition, motion correction in six directions to account for motion between volumes, spatial smoothing to minimize anatomical differences (FWHM = 4 mm), normalization to convert to percent signal change relative to the mean activation for the entire experiment, and high-pass filtering to remove slow trends (as in Leong et al., 2016).

#### Task behavior

Multilevel binary logistic regressions were carried out using the lme4 package (Version 1.11.1) (Bates et al., 2014) in R (Version 3.1.3). These models tested the effects of experimental conditions and age on trial-to-trial risky choices with intercepts that could vary across participants. This approach facilitated testing multiple conditions within each participant. The following was used to model the effects of age (varied between subjects; as a continuous variable), gamble type (varied within subjects; deviation coding used to compare each skew condition to the symmetric condition: Contrast 1 = positive skew > symmetric, Contrast 2 = negative skew > symmetric), and the interaction between these terms:

$$ {\displaystyle \begin{array}{l} ACCEPT={b}_{0j}+{b}_{1j}\left({age}_{ij}\right)+{b}_{2j}\left( gamble type\right)+{b}_{3j}\left( age\ x\  gamble type\right)+{e}_{ij}\\ {}{b}_{0j}={\beta}_{00}+{u}_{0j}\\ {}{b}_{1j}={\beta}_{10}+{u}_{1j}\ \end{array}} $$

Originally, previous outcome (loss, none, win) and its interactions, were included in the model (see online Supplementary Material). However, because diagnostic checks revealed collinearity with the gamble type regressor (VIF values >2), previous outcome and its interactions were removed. Taking out these predictors did not change the direction or significance of the results reported below. Follow-up bootstrapped statistics with 2,000 replications were conducted to verify each significant effect to confirm that outliers were not overly influencing the analysis.

#### Whole-brain analysis

Analyses focused on changes in brain activation during anticipation (that is, while participants viewed the gambles but before they made their choice). Based on extensive prior research that suggests that there are no adult age differences in neural activation following the receipt of gains versus losses (Samanez-Larkin et al., 2007; Samanez-Larkin et al., 2010; Samanez-Larkin et al., 2014), we did not predict age-related differences in neural activation in response to task outcomes. Following the procedures recommended by the AFNI group (Cox, Reynolds, & Taylor, 2016), voxel-wise statistical thresholds were set to *p* < .001, uncorrected, at the whole-brain level. The minimum cluster size of 18 contiguous, face-to-face 2.9-mm^3^ voxels for a cluster-level correction of *p* < .05 was estimated using the new autocorrelation function in AFNI’s 3dFWHMx and 3dClustSim. For each participant, the preprocessed time series data were analyzed with multiple regression models in AFNI. The models included two orthogonal contrasts of interest: (1) skewed versus symmetric (general skew), and (2) positive versus negative skew (valenced skew). Before inclusion in the regression models, these contrasts were convolved with a single gamma function to model the hemodynamic response. Subject-level regression models also included nine covariates of noninterest: residual motion (in six dimensions), white matter and cerebral spinal fluid time series, and polynomial trends across the experiment. For each contrast of interest, *T*-statistic maps were transformed to *Z* scores and spatially normalized by warping into Talairach space. The residual error time series from these subject-level models were used to estimate the noise smoothness values for cluster estimation. Statistical maps were then generated to examine three covariates of interest across the entire sample: (1) age, (2) positive-skew bias, and (3) the interaction between these two covariates. For follow-up verification of these analyses, regions of interest were specified around the significant clusters of activation that emerged in group analyses. Follow-up activity time-course analyses examined whether activation prior to choice, or anticipatory activation, (i.e., TRs 4 and 5 with a 4-s lag to account for the hemodynamic response) differed by gamble condition. Bootstrapped statistics with 2,000 replications were conducted to verify each significant effect to confirm that outliers were not overly influencing the analysis.

Study 2

A separate behavioral study was conducted with a much larger sample (*N* = 508) to evaluate the reliability of the age effects on choice. Detailed methods and results are presented in the Supplementary Materials.

## Results

### Study 1

#### Behavior

Overall, compared to symmetric gambles, participants were more likely to accept positively skewed gambles and more likely to reject negatively skewed gambles (see Table 2). An interaction of gamble type with age indicated that this effect was most pronounced in older adults, who were more likely to accept positively skewed than symmetric gambles and more likely to reject negatively skewed than symmetric gambles compared to younger adults (see Fig. 1b). Follow-up bootstrapped correlations confirmed that older adults were more willing accept positively skewed than symmetric gambles (*R =* .399, 95% CI [.047, .632 ]), but *did not* confirm that older adults were more likely to reject negatively skewed than symmetric gambles (*R* = −.135, 95% CI [−.468, .228]). To ensure that these age effects were not driven by outliers, we compared the difference between the proportion of positively skewed and negatively skewed gambles chosen by each participant. This individual difference measure—skew bias—varied from 1 to −1, with those who chose a higher proportion of positively skewed gambles (positive-skew bias) having positive scores and those who chose a higher proportion of negatively skewed gambles (negative-skew bias) having negative scores. While young participants varied widely on this measure, with similar numbers of young individuals showing negative and positive skew biases, almost all of the older participants displayed a positive skew bias (see Fig. 1c).

**Table 2:** Logistic regression models predicting risky choice

Variables	Comparison	Behavior
Intercept		.17 [−0.2, 0.53]
Skewness	Positive > Symmetric	**.17 [0.04, 0.3]**
	Negative > Symmetric	**−.54 [−0.68, −0.41]**
Age		−.27 [−0.75, 0.19]
Skewness by age	Positive > Symmetric × Age	**.62 [0.48, 0.76]**
	Negative > Symmetric × Age	**−.41 [−0.56, −0.27]**
AIC		2,646.9
BIC		2,698.5
Pseudo *R* ^2^		.34

*Note*. Unstandardized betas (and 95% confidence interval) reported. Participants modeled as random effects. Significant fits highlighted in bold

#### Whole-brain analyses

**Fig. 2:**
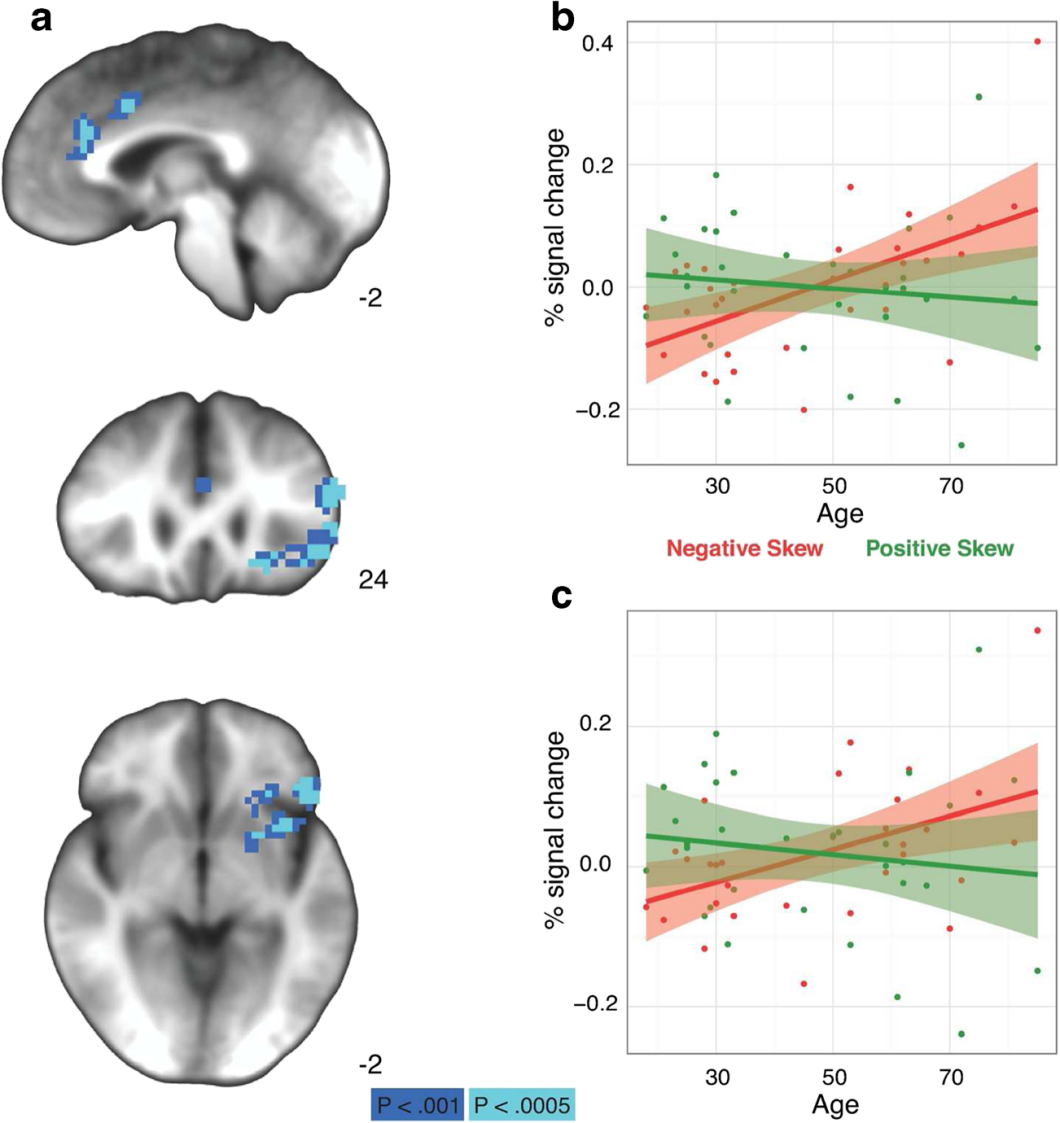
Neural age by valence interaction during skewed gambling task. Top: Right anterior cingulate. Bottom: Left inferior/middle frontal gyrus. Middle: Both regions. **a** Clusters where there was a significant Age × Valence interaction (Left=right). **b–c** Percentage signal change in each region over age (in years) split by positive and negative skew trials. (Color figure online)

First-level general linear models quantified differences between skewed versus symmetric trials (general skew) and positively skewed versus negatively skewed trials (valenced skew) across the brain. Second-level regression analyses examined individual differences by age and behavioral bias scores on the effects of general skew and valenced skew. Both main and interaction effects of age and behavioral bias were included together in second-level models because of the behavioral interaction between age and skew bias. Whole-brain analyses revealed significant age differences in anticipatory neural activity related to both skew and valence (see Tables 3 and 4). In a large cluster in the left frontal gyrus and the left anterior insula (AIns), age was associated with *increased* activation in anticipation of negatively skewed gambles and *reduced* activation for positively skewed gambles, and a similar interaction was evident in the right anterior cingulate (see Fig. 2). Follow-up bootstrapped correlations of age on the average neural activity during the anticipatory period (TRs 4 and 5) in these volumes of interest (VOIs) for each contrast of interest confirmed that both of these interactions were driven by age-related increase in activation in anticipation of negatively skewed gambles (left AIns: *R* = .473 95% CI [0.193, 0.714]; right anterior cingulate: *R* = .566 [0.247, 0.742]).

**Table 3:** Effects on skewed versus symmetric gambles on brain activity

Region	R	A	S	Z	# Voxels
Age					
Right precentral gyrus	28	−11	55	−4.32	55
Right inferior frontal gyrus	39	27	14	−5.10	34
Right inferior occipital gyrus	33	−80	−6	−4.43	32
Left fusiform gyrus	−28	−60	−9	−4.05	28
Right culmen	33	−51	−15	−4.51	19

**Table 4:** Effects on positively skewed versus negatively skewed gambles on brain activity

Region	R	A	S	Z	# Voxels
Age					
Left inferior/middle frontal gyrus	−51	24	20	−5.10	238
Right anterior cingulate	1	30	23	−4.64	47
Left inferior frontal gyrus/insula	−25	24	−6	−4.49	39
Left cingulate gyrus	−4	10	35	−4.25	32
Left central precuneus	−42	−5	38	−4.20	24
Left superior temporal gyrus	−57	−43	6	−3.74	19
Bias					
Left cingulate gyrus	−4	12	32	4.90	81
Left caudate/nucleus accumbens	−10	10	−6	4.66	71
Right caudate	7	15	0	4.59	70
Left insula	−30	−19	17	4.79	52
Right insula	39	−8	14	4.25	32
Age × Bias					
Right insula	39	−22	14	4.95	129
Right inferior frontal gyrus	51	24	6	4.71	46
Left cingulate gyrus	−4	15	29	5.14	46
Right inferior frontal gyrus	30	18	−15	4.79	27
Right posterior cingulate	25	−51	20	4.59	22
Right superior temporal gyrus	51	18	−15	5.28	20

**Fig. 3:**
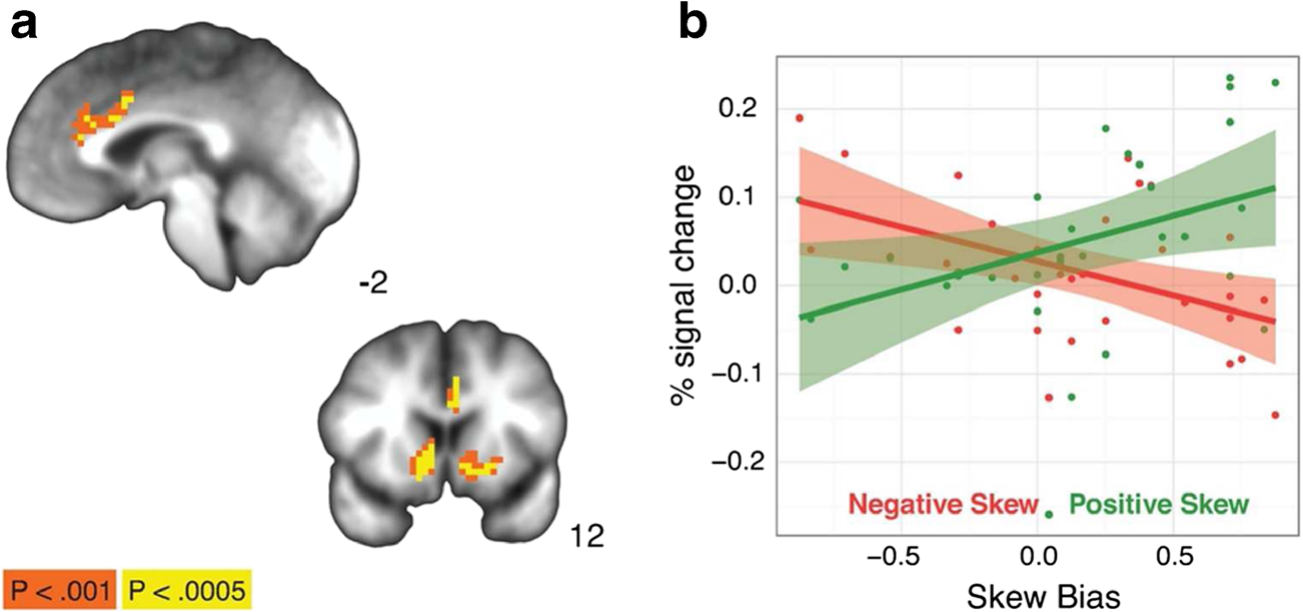
Neural skew bias by valence interaction during skewed gambling task. Top: Left cingulate gyrus. Bottom: Bilateral caudate. **a** Clusters where there was a significant Bias Score × Valence interaction (Left=right). **b** Percentage signal change in the bilateral caudate clusters over skew biasscore split by positive and negative skew trials. (Color figure online)

Individual differences in the behavioral measure of positive skew bias from the task were also related to individual differences in anticipatory brain activity related to skew valence. In clusters along the cingulate gyrus, as well as bilateral regions of the caudate and posterior insula, a behavioral positive-skew bias was associated with *increased* activation for positively skewed gambles and *decreased* activation for negatively skewed gambles (see Fig. 3). The reverse was true for individuals with a negative-skew bias; in these regions, negative-skew bias was associated with *decreased* activation for positively skewed gambles and *increased* activation for negatively skewed gambles. Follow-up bootstrapped correlations of positive-skew bias on the anticipatory activation in the bilateral caudate VOI confirmed that this interaction was driven by both a positive-skew-bias-related *increase* in anticipator activation for positively skewed gambles (*R* = .384, 95% CI [0.058, 0.62]) and a bias-related *decrease* in anticipatory activation for negatively skewed gambles (*R* = −0.467, 95% CI [−0.722, −0.129]).

**Fig. 4:**
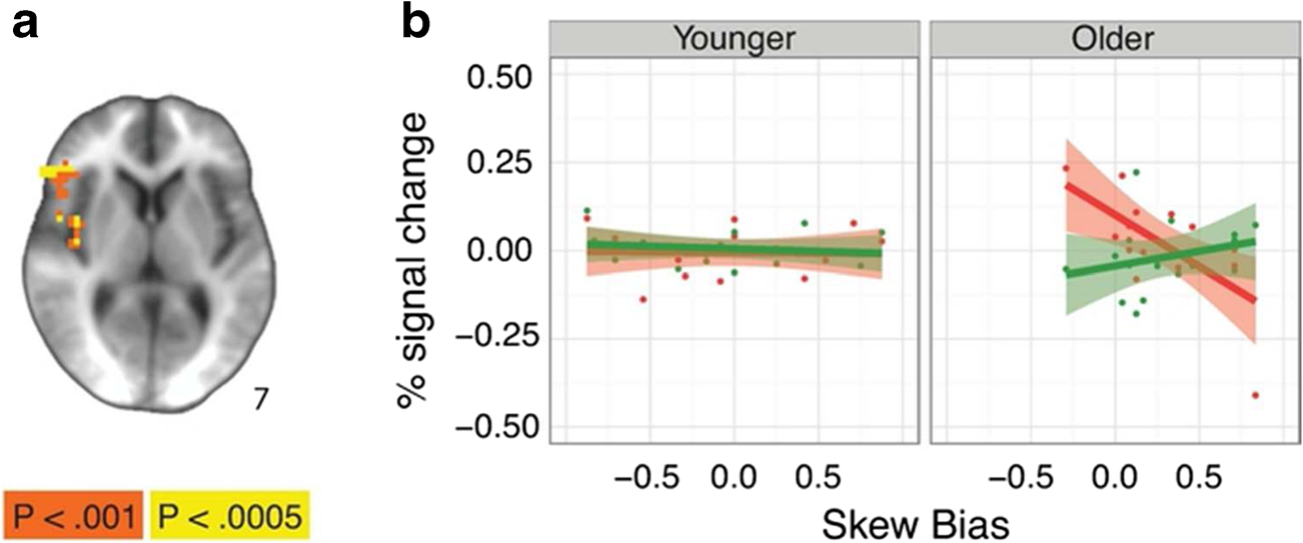
Neural Age × Skew Bias × Valence interaction during skewed gambling task. **a** Clusters where there was a significant Age × Bias Score × Valence interaction (Left=right). **b** Percentage signal change in the right inferior frontal gyrus cluster over skew bias score split by positive and negative skew trials in younger (left) and older (right) adults. Age groups created by conducting a median split by age for illustrative purposes only. (Color figure online)

Finally, there was a significant interaction between age and behavioral skew bias on skew-related anticipatory brain activity in several regions (see Table 4). Follow-up bootstrapped regression of age and positive-skew bias on anticipatory activity in the right inferior frontal gyrus VOI confirmed that this interaction was significant (*b* = 0.01, 95% CI [0.003, 0.017]). Within older adults, individuals showed increased activity in the right inferior frontal gyrus on trials where they were more likely to accept the gamble: those with a positive-skew bias showed greater activity on positively skewed trials, while those with a negative-skew bias showed greater activity on negatively skewed trials. Follow-up bootstrapped correlations of bias on anticipatory activity in right inferior frontal gyrus VOI in older adults confirmed that this interaction was driven by a bias-related increase in activation in anticipation of negatively skewed gambles (*R* = .664, 95% CI [−0.857, −0.298]). These associations were not present in younger adults (see Fig. 4).

### Study 2

#### Behavior

Consistent with the behavioral results from Study 1, participants in Study 2 were more likely to *accept* positively skewed (compared to symmetric) gambles and more likely to *reject* negatively skewed (compared to symmetric) gambles (see Table S2 in the Supplementary Material). Also similar to Study 1, in Study 2 an interaction of gamble type with age indicated that this trend was most pronounced in older adults, who were more willing to accept positively skewed than symmetric gambles (see Fig. 4b). Full results are presented in the Supplementary Materials.

## Discussion

This study investigated individual and adult age differences in choice and neural activity during skewed financial risk-taking. Controlling for age, behavioral and neural results were consistent with previous findings in young adults. Specifically, participants showed greater acceptance of positively skewed gambles relative to negatively skewed gambles, consistent with similar patterns in the economics (Åstebro, Mata, & Santos-Pinto, 2009), psychology (Hertwig & Erev, 2009), and neuroeconomics (Burke & Tobler, 2011; Wu et al., 2011) literature.

Although positively skewed and negatively skewed gambles were equivalent in *expected* value, many participants revealed either an increased preference for positively skewed gambles, or a decreased preference for negatively skewed gambles, or both. This suggests that many participants placed relatively greater *subjective* value on positively skewed than on negatively skewed gambles. Independent of age, we found that activity in regions typically associated with subjective reward valuation (Bartra, McGuire, & Kable, 2013) and risk (Knutson & Huettel, 2015)—including the medial frontal cortex, caudate, and insular cortex—were associated with behavioral preferences for skewed gambles. Consistent with a subjective valuation account (Acikalin, Gorgolewski, & Poldrack, 2017; Bartra et al., 2013; Clithero & Rangel, 2013), these regions were more active on trials where participants were more likely to accept the gamble (see Fig. 3). Although the magnitude and direction of previously reported effects in these cortical and striatal regions has varied across studies, both NAcc and AIns activity were previously associated with processing of skewed gambles (Burke & Tobler, 2011; Symmonds, Wright, Bach, & Dolan, 2011; Wright, Symmonds, Morris, & Dolan, 2013; Wu et al., 2011; Wu et al., 2012). Recruitment of these regions is also consistent with a broader literature on anticipatory affect and motivation (Knutson et al., 2014). Specifically, ventral striatal activity (including NAcc activity) often predicts approach behavior (Knutson & Greer, 2008). Results of trial-level logistic regression analyses (see Table S1, Fig. S1 in the Supplementary Material) further supported these claims, showing that increased activity in the NAcc predicted increased gamble acceptance.

Inclusion of an adult life-span sample allowed us to test the generality of these associations over adult development. The results revealed that differences in behavioral preferences for positively skewed over symmetric gambles increased with age, since acceptance rates for positively skewed gambles increased with age. Follow-up analyses confirmed that these trends were not driven by outliers or an artifact of averaging, since almost all older adults demonstrated a positive-skew bias. Further, we replicated these behavioral results in a large online study (*N* = 508), where again we found an age-related increase in acceptance of positively skewed gambles relative to symmetric gambles (see Table S2, Fig. S4b in the Supplementary Material). These findings might begin to clarify some inconsistencies reported in previous studies of the effects of age on risk preferences, since prior studies have not accounted for the skewness of risky choices. When provided with explicit probabilities, people tend to overweight the magnitude of low probability events (Hertwig & Erev, 2009), which might bias older adults even more than younger adults.

The predicted but novel age-related increase in a bias toward positively skewed gambles is consistent with the age-related positivity effect (Mather & Carstensen, 2005). Prior studies examining how this developmental trend influences choice have focused on how positivity shapes attention to, recall of, and satisfaction with decisions (Kim, Healey, Goldstein, Hasher, & Wiprzycka, 2008; Löckenhoff & Carstensen, 2007; Mather & Johnson, 2000) but not the actual choices made by participants. Despite the fact that all decision options used in this study were equated for expected value and variance, older adults preferred positively skewed gambles more than their younger counterparts. This provides direct evidence for an age-related positivity effect in *risky choice,* consistent with studies of attention, memory recall, and choice satisfaction. The findings suggest that an increased focus on positive incentives may bias choice and could increase susceptibility to fraudulent investments and lotteries in real life. We also hypothesized that age may have been related to a decreased preference for negatively skewed gambles but this was not a reliable effect in Study 1 and a nonsignificant effect in Study 2.

Age was also associated with activity in the left inferior frontal gyrus (IFG), AIns, and anterior cingulate cortex (ACC), regions commonly associated with executive function and cognitive control (Botvinick & Braver, 2015). During our task, these regions showed greater activity for negatively skewed gambles and less activity for positively skewed gambles in older adults. This pattern of activity opposed that observed in behavior (see Fig. 1) and may partially indicate that these lateral cortical systems inhibited the activity of subcortical systems (Daw, Niv, & Dayan, 2005). This suggests that compared to young adults, older adults may be engaging more cognitive control resources on negatively skewed trials, leading them to reject negatively skewed gambles more frequently. Using reverse inference in Neurosynth to examine the relative selectivity of the regions showing age differences (Yarkoni, Poldrack, Nichols, Van Essen, & Wager, 2011), we found that the more medial IFG cluster [30, 18, −15] was strongly associated with “executive function,” *Z* = 5.97. However, because this interpretation depends on reverse inference, but not experimental manipulation, it remains an open question for future research.

Many studies of aging show that older adults recruit broader neural networks in cognitive tasks. While the left IFG was recruited by both age groups when considering skewed gambles, only older adults also engaged the right IFG. In particular, the right IFG was more active on trials where older adults were more likely to accept the gamble. This pattern is consistent with a general theme of increased bilateral recruitment of prefrontal regions in older adults (Cabeza & Dennis, 2012; Reuter-Lorenz & Park, 2014). “Hyperactivation” has been interpreted as compensatory, such that older adults recruit more neural resources to counteract age-related declines in other neural circuits. The risky gambling task lacks objective performance measures, however, so the observed right IFG activity does not meet formal criteria for compensation (Cabeza & Dennis, 2012). Overall, both the main effects of and interactions with age on neural activity were not predicted. Future research will need to clarify how and whether these systems interact to influence skewed risk-taking differentially across adulthood.

Although little research has focused on skewed risk preferences, many major financial choices in everyday life necessarily invoke skewed risks. For example, financial fraud (where individuals accept highly positively skewed risks) is a significant and growing problem for people of all ages. While some evidence suggests that older adults might not necessarily be more susceptible to fraud (Ross et al., 2014), the consequences of being financially defrauded in older age are much more severe, since older individuals have more to lose but limited time and marketable labor resources to recover from a major financial loss (MetLife Mature Market Institute, 2011). Both researchers and policy makers are interested in identifying and reducing susceptibility to victimization by financial fraud. These findings imply that age-related preference for skewed risks assessed in the laboratory might provide some index of fraud susceptibility in everyday life. We conducted additional exploratory analyses to assess possible associations between skew sensitivity and fraud susceptibility. Although behavioral choices of skewed gambles were not directly related to fraud susceptibility, neural measures did show some association with perceived vulnerability to fraud (see online Supplementary Material). Although this represents a single study on a relatively small sample, the results suggest that neural measures might enable identification of vulnerable individuals, which could bolster prevention efforts before fraud occur (Scheibe et al., 2014).

## Electronic supplementary material

ESM 1 (DOCX 1104 kb)
