# Supplementary material for: Individual differences in skewed financial risk-taking across the adult life span
Source: Cogn Affect Behav Neurosci. 2017 Oct 23;17(6):1232–41. doi: 10.3758/s13415-017-0545-5 (PMC5709503; doi:10.3758/s13415-017-0545-5)
Supplement: Supplementary file 1 — (DOCX 1104 kb) [file 13415_2017_545_MOESM1_ESM.docx]

**Supplementary Material**

**Study 1: Volume of interest (VOI) analyses and fraud susceptibility**

The binary logistic regression models used to examine age effects on choice behavior in the manuscript were extended by adding activity from a priori volumes of interest (NAcc and AIns) to examine the additional unique effects of brain activity on choice. For each VOI, mean activity prior to each choice (TRs 4 and 5) was entered into the model to predict subsequent choice for each trial. Adding brain activity from predicted regions (NAcc and AIns) to the logistic regression models significantly increased the model’s prediction of risky choice (see Table S1, w/Brain Activity column). Specifically, greater bilateral NAcc activity predicted gamble acceptance, while greater bilateral AIns activity predicted gamble rejection. Both of these effects were qualified, however, by interactions with age (see Figs.S1 and S2), such that the correlation between brain activity and risky choice was reduced in older adults.

Age differences were also found in associations of brain activity with risky choice. Compared to young adults, the association between activity in the NAcc and AIns with skewed risk-taking was diminished in older adults. Although the reduced association of age with AIns activity was expected, the reduced association of age with NAcc activity was not. Based on prior research showing similar NAcc responses across adulthood (Samanez-Larkin et al., 2014; Samanez-Larkin et al., 2007; Spaniol, Bowen, Wegier, & Grady, 2015), we predicted that the relationship between NAcc activity and choice behavior would be preserved with age. Also, these results alone cannot account for increased preference for positive skew gambles. While older adults demonstrated greater acceptance of positively skewed gambles, there was no concordant increase in NAcc activity for older adults.

Anterior insula (AIns) activity is often associated with avoidance behavior (Knutson & Greer, 2008), and our finding that increasing AIns activity predicts increased gamble rejection in VOI/logistic-regression analyses is consistent with this. However, our whole-brain analyses revealed that more posterior regions of the insula were more active on trials where participants were more likely to accept, instead of reject, the gamble. This functional distinction is consistent with a growing literature parcellating the insula into two subregions: the ventral-anterior insula and the dorsal-posterior insula (Cauda et al., 2011; Deen, Pitskel, & Pelphrey, 2011; Leong, Pestilli, Wu, Samanez-Larkin, & Knutson, 2016; Nanetti, Cerliani, Gazzola, Renken, & Keysers, 2009; Taylor, Seminowicz, & Davis, 2009). Connectivity studies suggest the anterior insula is part of a network of regions, including the anterior cingulate cortex and other subcortical regions associated with responses to pain and emotional stimuli. On the other hand, the posterior insula is part of a different network of regions including the midcingulate and motor cortices that has been associated with action selection and cognitive control (Taylor et al., 2009). Furthermore, these two insula-cingulate networks are thought to work together, with the more anterior network feeding information to the more posterior network (Flynn, 1999; Singer, Critchley, & Preuschoff, 2009). All of this together suggests that the activity we observed in the anterior insula might reflect an initial emotional response to skewed gambles, whereas activity in more posterior regions of the insula might reflect a higher level plan of action.

Self-report questionnaires assessed participants’ own perceived fraud susceptibility. Participants were asked about their ability to detect fraud and resist persuasion, including: (a) “How likely are you to make a fraudulent investment?”, (b) “How able are you to detect a fraudulent investment?”, and (c) “How able are you to resist high-pressure sales tactics when buying investments?” Participants responded using a 7-point scale ranging from *not at all likely/able to detect/able to resist* to *very likely/able to detect/able to resist*.

To examine whether choice and neural activity were associated with trial-to-trial choices and real-life financial outcomes, partial correlations between choice behavior, brain activity during anticipation of risky choice, and fraud susceptibility were calculated after controlling for age. These exploratory analyses revealed that AIns activity during presentation of negatively skewed gambles was negatively associated with individual differences in self-reported fraud susceptibility (Question a; *r* = −0.451 95% CI [−0.69, −0.12]; see Fig. S2c). No other behavioral or neural predictors showed significant associations with fraud susceptibility. Interestingly, the three self-report questions related to fraud susceptibility were not significantly correlated with each other, *r*s < .20, *p*s > .20.

| **Table S1**  Logistic regression models predicting risky choice | | | |
| --- | --- | --- | --- |
| Variables | Comparison | Behavior | w/Brain Activity |
| Intercept |  | 0.17 [−0.2, 0.53] | 0.11 [−0.27, 0.49] |
| Skewness | Positive > Symmetric | **0.17 [0.04, 0.3]** | 0.1 [−0.04, 0.24] |
|  | Negative > Symmetric | **−0.54 [−0.68, −0.41]** | **−0.54 [−0.68, −0.4]** |
| Age |  | −0.27 [−0.75, 0.19] | −0.26 [−0.77, 0.23] |
| NAcc |  |  | **2.19 [1.77, 2.62]** |
| AIns |  |  | **−0.58 [−1.02, −0.15]** |
| Skew × Age | Positive × Age | **0.62 [0.48, 0.76]** | **0.67 [0.53, 0.82]** |
|  | Negative × Age | **−0.41 [−0.56, −0.27]** | **−0.42 [−0.57, −0.27]** |
| Neural Activity × Age | NAcc × Age |  | **−0.74 [−1.13, −0.35]** |
|  | AIns × Age |  | **0.72 [0.34, 1.1]** |
| AIC |  | 2,646.9 | 2510.9 |
| BIC |  | 2,698.5 | 2585.3 |
| Pseudo *R*^2^ |  | .34 | .41 |
| Model χ^2^ |  |  | 144.09*** |
| *Note*. Unstandardized betas (and 95% confidence interval) reported. Subjects modeled as random effects. Significant fits highlighted in bold. NAcc = nucleus accumbens (bilateral); AIns = anterior insula (bilateral) | | | |

**Fig. S1** Nucleus accumbens (NAcc) activity during the gambling task. **a** NAcc region of interest. **b** Probability of gamble acceptance over percent signal change in the NAcc by median-split age group (controlling for other predictors in Model 2)

**Fig. S2** Anterior insula (AIns INS) activity during the gambling task. **a** AIns region of interest. **b** Probability of gamble acceptance over percentage signal change in the INS by median-split age group (controlling for other predictors in Model 2). **c** Self-reported likelihood of fraud over percentage signal change in the AIns

**Study 2 (online): Methods and results**

Five hundred and eight participants (age: *M* = 48.62 years, *SD* = 17.02, range: 20–81 years) were recruited for an online study using Qualtrics panels. Screening questions were used to exclude people with a history of psychiatric illness or prior head injury. Age and gender quotas were used to ensure the sample included equal numbers of men and women in each age decade. Participants completed a brief demographic questionnaire, a brief skewed gambling task (more details below), and a brief risky decision-making task (not reported here). Total survey time was approximately 10 minutes.

As in the neuroimaging study, during the skewed gambling task, participants chose between a safe, certain amount ($0; reject the gamble) and a risky, uncertain gamble (accept the gamble). In contrast to the neuroimaging study, the gambles did not play out and the participants received no feedback after their choices. The task was also not incentive compatible in that subjects were not paid according to their choices; choices were made hypothetically. There were three gamble types: positively skewed, negatively skewed, and symmetric gamble. For mixed gambles, the exact same dollar amounts and probabilities for each gambles type were used in this study (see Fig. S3b). However, unlike the neuroimaging study, participants only saw each mixed gamble type three times (for a total of nine mixed gambles).

In addition to the mixed gambles described above, participants also saw three “gain” and three “loss” gambles (see Fig. S3b) to examine whether age differences were due to potential differences in framing effects.

In the first wave of data collection (*N* = 110), participants made choices in the following order: three mixed gambles, three gain gambles, three mixed gambles, three loss gambles, three mixed gambles (15 gambles total). Within each set of gambles the participants saw one positively skewed gamble, one negatively skewed gamble, and one symmetric gamble. Because of concerns about order effects, for the second wave of data collection (*N* = 398), participants made choices about all three sets of mixed gambles first (nine gambles), and then made choices about a set of gain gambles and a set of loss gambles (15 gambles total). The presentation of gambles was randomized within each set of three gambles. There was no significant difference in gamble acceptance between waves, so the data from both waves is grouped together below.

Similar to the main text, multilevel binary logistic regressions were carried out using the lme4 package in R. The following models were used to model the effects of age (varied between subjects; as a continuous variable), gamble type (varied within subjects; deviation coding used to compare each skew condition to the symmetric condition: Contrast 1 = Positive Skew > Symmetric, Contrast 2 = Negative Skew > Symmetric), domain (varied within subjects; deviation coding used to compare each domain, gain or loss, to mixed gambles: Contrast 1 = Gain > Mixed; Contrast 2 = Loss > Mixed), and the two-way interactions between all terms and age on gamble acceptance:

**Model 1**

$$ACCEPT= b_{0j}+ b_{1j}\left( {age}_{ij} \right)+ b_{2j}\left( gamble type \right)+ b_{3j}\left( gamble domain \right)+b_{4j}\left( age x gamble type \right)+b_{5j}\left( age x gamble domain \right)+ e_{ij}$$

$$b_{0j}= \beta_{00}+ u_{0j}$$

$b_{1j}= \beta_{10}+ u_{1j}$.

We also tested a model similar to that from the neuroimaging analysis on the data from the mixed trials only:

**Model 2**

$$ACCEPT= b_{0j}+ b_{1j}\left( {age}_{ij} \right)+ b_{2j}\left( gamble type \right)+b_{4j}\left( age x gamble type \right)+ e_{ij}$$

$$b_{0j}= \beta_{00}+ u_{0j}$$

$b_{1j}= \beta_{10}+ u_{1j}$.

Compared to mixed gambles, participants were more likely to *accept* gambles in the gain domain and more likely to *reject* gambles in the loss domain (see Table S2, Model 1, Fig. S3a). Consistent with the results from our imaging data set, participants were more likely to *accept* positively skewed (compared to symmetric) gambles and more likely to *reject* negatively skewed (compared to symmetric) gambles (see Table S2). Furthermore, an interaction of gamble type with age indicated that this trend was most pronounced in older adults, who were more willing to accept positively skewed than symmetric gambles (see Fig. S4b). These last two trends were significant when we limited the analysis to only mixed gambles (see Table S2, Model 2).

| **Table S2**  Logistic regression models predicting risky choice | | | |
| --- | --- | --- | --- |
| Variables | Comparison | Model 1 | Model 2 |
| Intercept |  | **0.28 [0.17, 0.38]** | **0.4 [0.24, 0.57]** |
| Skewness | Positive > Symmetric | **0.31 [0.22, 0.39]** | **0.56 [0.46, 0.67]** |
|  | Negative > Symmetric | −**0.36 [−0.44, −0.27]** | **−0.57 [−0.67, −0.47]** |
| Domain | Gain > Mixed | **0.08 [−0.03, 0.19]** |  |
|  | Loss > Mixed | **0.25 [0.18, 0.32]** |  |
| Age |  | −0.29 [−0.36, −0.22] | 0.11 [−0.05, 0.28] |
| Skewness × Age | Positive > Symmetric × Age | **0.09 [0.02, 0.16]** | **0.21 [0.1, 0.32]** |
|  | Negative > Symmetric × Age | 0.03 [−0.04, 0.1] | 0.01 [−0.09, 0.12] |
| Domain × Age | Gain > Mixed × Age | −0.02 [−0.1, 0.07] |  |
|  | Loss > Mixed × Age | 0.02 [−0.07, 0.11] |  |
| AIC |  | 9,650.8 | 5,362.9 |
| BIC |  | 9,741.0 | 5,420.7 |
| Pseudo *R*^2^ |  | .26 | .47 |
| *Note*. Unstandardized betas (and 95% confidence interval) reported. Participants modeled as random effects. Significant fits highlighted in bold | | | |

| a) |  |  |  |
| --- | --- | --- | --- |
| 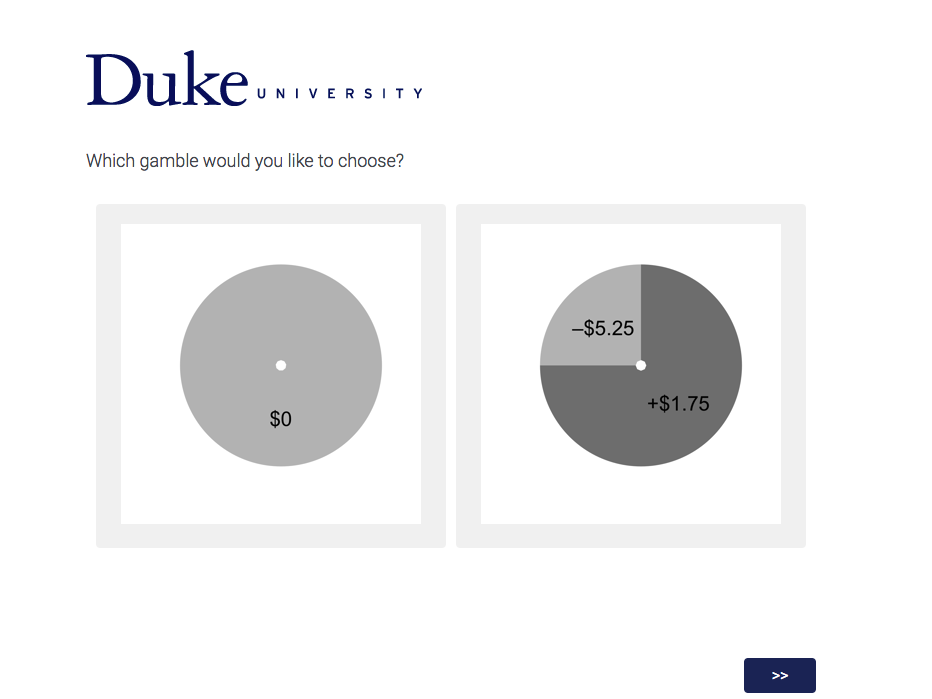 | | | |
| b) |  |  |  |
|  | **Mixed** | **Gain** | **Loss** |
| Certain option | 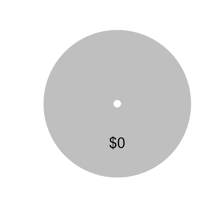 | 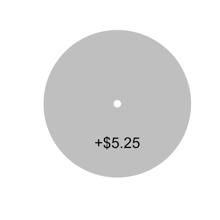 | 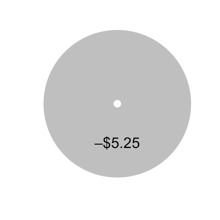 |
| or | | | |
| Positively skewed | 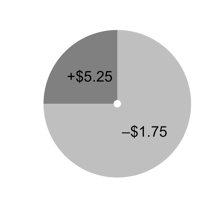 | 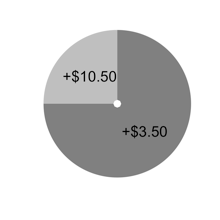 | 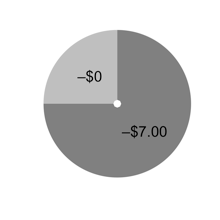 |
| Symmetric | 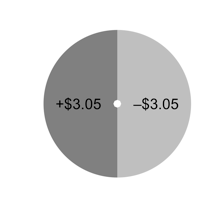 | 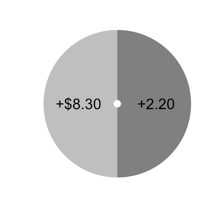 | 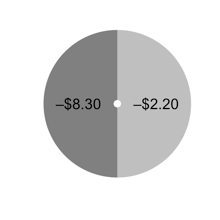 |
| Negatively skewed | 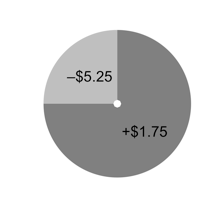 | 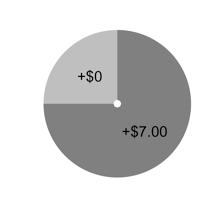 | 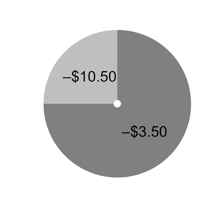 |

**Fig. S3** Online skewed gambling task. **a** Trial structure of online gambling task. **b** All combinations of skew (positively skewed, symmetric, and negatively skewed) with gambling frame (mixed, gain, or loss)

**Fig. S4** Online skewed gambling behavior. **a** Proportion of trials where the gamble was accepted by gamble domain (gain, loss, or mixed) and gamble type (positive skew, symmetric, negative skew). **b** Proportion of mixed trials where the gamble was accepted over age by gamble type

**Study 1: Previous outcome**

Multilevel binary logistic regressions examined the influence of previous outcome on gamble acceptance rates in R. The following model was used to model the effects of age, gamble type, previous outcome (varied within subjects; deviation coding used to compare wins and losses to no outcome, no gamble: Contrast 1 = Loss > None, Contrast 2 = Win > None, and all possible interactions between these terms:

$$ACCEPT= b_{0j}+ b_{1j}\left( {age}_{ij} \right)+ b_{2j}\left( gamble type \right)+ b_{3j}\left( previous outcome \right)+ b_{4j}\left( age x gamble type \right)+ b_{5j}\left( age x previous outcome \right)+ b_{6j}\left( gamble type x previous outcome \right)+ b_{7j}\left( age x gamble type x previous outcome \right)+ e_{ij}$$

$$b_{0j}= \beta_{00}+ u_{0j}$$

$$b_{1j}= \beta_{10}+ u_{1j} .$$

Overall, compared to symmetric gambles, participants were more likely to accept positively skewed gambles but more likely to reject negatively skewed gambles (see Table S3). An interaction of gamble type with age indicated that this trend was most pronounced in older adults, who were more willing to accept positively skewed than symmetric gambles but more likely to reject negatively skewed than symmetric gambles compared to younger adults. Overall, compared to no-gamble trials, participants were more willing to accept gambles after a loss (see Table S3). However, an interaction indicated the varied for negatively skewed gambles. Compared to no-gamble trials, participants were *more* likely to accept negatively skewed gambles (compared to symmetric) following a win and *less* likely to accept negatively skewed gambles (compared to symmetric) after a loss (see Fig. S5).

| **Table S3**  Logistic regression models predicting risky choice | | |
| --- | --- | --- |
| Variables | Comparison | Behavior |
| Intercept |  | 0.2 [−0.16, 0.54] |
| Skewness | Positive > Symmetric | **0.19 [0.05, 0.33]** |
|  | Negative > Symmetric | **−0.56 [−0.7, −0.42]** |
| Previous outcome | Loss > None | **0.23 [0.08, 0.38]** |
|  | Win > None | −0.05 [−0.2, 0.1] |
| Age |  | −0.24 [−0.71, 0.2] |
| Skewness × Previous Outcome | Positive > Symmetric × Loss > None | 0.14 [−0.06, 0.35] |
|  | Negative > Symmetric × Loss > None | **−0.41 [−0.62, −0.21]** |
|  | Positive > Symmetric × Win > None | −0.15 [−0.36, 0.06] |
|  | Negative > Symmetric × Win > None | **0.34 [0.14, 0.54]** |
| Skewness × Age | Positive > Symmetric × Age | **0.62 [0.47, 0.77]** |
|  | Negative > Symmetric × Age | **−0.38 [−0.53, −0.24]** |
| Previous Outcome × Age |  | 0.03 [−0.13, 0.19] |
|  |  | 0.07 [−0.08, 0.23] |
| Skewness × Previous Outcome × Age | Positive > Symmetric × Loss > None × Age | −0.08 [−0.3, 0.14] |
|  | Negative > Symmetric × Loss > None × Age | 0.16 [−0.05, 0.38] |
|  | Positive > Symmetric × Win > None × Age | 0.09 [−0.12, 0.31] |
|  | Negative > Symmetric × Win > None × Age | −0.01 [−0.22, 0.21] |
| AIC |  | 2,635.1 |
| BIC |  | 2,755.4 |
| Pseudo *R*^2^ |  | .34 |
| *Note*. Unstandardized betas (and 95% confidence interval) reported. Participants modeled as random effects. Significant fits highlighted in bold | | |

**Fig. S5** Skewed gambling behavior. **a** Proportion of trials where the gamble was accepted over the outcome of the previous trial (loss, win, none) by gamble type (negative skew, positive skew, symmetric)

**Study 1: Learning**

Here, multilevel *logistic growth curve* analysis was carried out using the lme4 package in R. To test both linear and quadratic effects of time, trial was transformed into two orthogonal polynomial terms (representing linear and quadratic effects of trial). The following model was used to model the effects of age, gamble type (contrasts as above), linear and quadratic effects of trial (varied within condition; sequence from 1 to 24), and all possible interactions between these terms:

$$ACCEPT= b_{0j}+ b_{1j}\left( {age}_{ij} \right)+ b_{2j}\left( gamble type \right)+ b_{3j}\left( linear \right)+ b_{4j}\left( quadratic \right)+ b_{5j}\left( age x gamble type \right)+ b_{6j}\left( age x linear \right)+ b_{7j}\left( age x quadratic \right)+ b_{8j}\left( gamble type x linear \right)+ b_{9j}\left( gamble type x quadratic \right)+ b_{10j}\left( age x gamble type x linear \right)+b_{11j}\left( age x gamble type x quadratic \right)+ e_{ij}$$

$$b_{0j}= \beta_{00}+ u_{0j}$$

$$b_{1j}= \beta_{10}+ u_{1j}$$

$$b_{3j}= \beta_{30}+ u_{3j}$$

$$b_{4j}= \beta_{40}+ u_{4j}.$$

Visual inspection of the data (Figure S6) showed that experience had the strongest influence on symmetric gambles, with participants in both age groups accepting symmetric gambles less with experience. Age differences with experience are also visible in the data. Young adults accepted positively skewed and negatively skewed gambles equally often, and this pattern was relatively consistent with experience. Older adults, on the other hand, accepted positively skewed gambles more than negatively skewed gambles, but the magnitude of this difference diminished with experience.

Statistical tests support these observations. The statistical model was significantly improved by adding linear, *χ*^2^(9) = 99.53, *p* < .001, and quadratic, *χ*^2^(10) = 20.11, *p* = .028, terms. Similar to the analyses reported in the main text, the largest effects were main effects of gamble type, as well as interactions between age and gamble type (see Table S4). Additionally, there were significant interactions between trial (both linear and quadratic terms), positively skewed (vs. symmetric) gambles, and age. Initially, younger adults were *less* likely to accept positively skewed gambles than symmetric gambles (see Fig. S6). However, with experience, younger adults accepted positively skewed gambles and symmetric gambles equally often. Older adults, on the other hand, initially accepted positively skewed and symmetric gambles equally often. However, with experience, they older adults accepted positively skewed gambles *more* often than symmetric gambles.

| **Table S4**  Logistic growth curve models predicting risky choice | | |
| --- | --- | --- |
| Variables | Comparison | Behavior |
| Intercept |  | 0.19 [−0.13, 0.52] |
| Age  Gamble Type |  | −0.18 [−0.66, 0.13] |
|  | Positive > Symmetric | **0.19 [0.05, 0.31]** |
|  | Negative > Symmetric | **−0.58 [−0.72, −0.46]** |
| Linear Trial  Quadratic Trial |  | −1.55 [−3.09, 0.23] |
|  |  | −0.31 [−1.27, 0.72] |
| Age × Gamble Type | Positive × Age | **0.66 [0.53, 0.8]** |
|  | Negative × Age | **−0.44 [−0.57, −0.32]** |
| Age × Linear |  | 0.17 [−1.09, 1.98] |
| Age × Quadratic |  | 0.79 [−0.38, 1.73] |
| Linear × Gamble Type | Positive × Linear | **1.85 [0.84, 3.03]** |
|  | Negative × Linear | 0.83 [−0.18, 1.88] |
| Quadratic × Gamble Type | Positive × Quadratic | **−1.36 [−2.59, −0.34]** |
|  | Negative × Quadratic | **1.15 [0.01, 2.16]** |
| Age × Gamble Type × Linear | Age × Positive × Linear | **−1.25 [−2.41, −0.21]** |
|  | Age × Negative × Linear | 0.66 [−0.41, 1.71] |
| Age × Gamble Type × Quadratic | Age × Positive × Quadratic | **−1.45 [−2.51, −0.29]** |
|  | Age × Negative × Quadratic | 1.03 [−0.03, 2.24] |
| AIC |  | 2,600.2 |
| BIC |  | 2,761.0 |
| Pseudo *R*^2^ |  | .451 |
| *Note*. Unstandardized betas (and 95% confidence interval) reported. Participants modeled as random effects. Significant fits highlighted in bold | | |

 **Fig. S6** Learning. Proportion of trials where gamble was accepted over trial by gamble in older (top) and younger (bottom) adults. Age groups created by conducting a median split by age for illustrative purposes only. Linear trends displayed on left and quadratic trends displayed on right

**Study 1: Response times**

Here, multilevel *linear* regressions were carried out using the lme4 package in R. These models tested the effects of experimental conditions and age on trial-to-trial response times with intercepts that could vary across participants. The following model was used to model the effects of age, gamble type, acceptance (varied within subjects; dummy coded: Reject = 0, Accept = 1) and all possible interactions between these terms:

$$RT= b_{0j}+ b_{1j}\left( {age}_{ij} \right)+ b_{2j}\left( gamble type \right)+ b_{3j}\left( accept \right)+ b_{4j}\left( age x gamble type \right)+ b_{5j}\left( age x accept \right)+ b_{6j}\left( gamble type x accept \right)+ b_{7j}(age x gamble type x accept)+ e_{ij}$$

$$b_{0j}= \beta_{00}+ u_{0j}$$

$$b_{1j}= \beta_{10}+ u_{1j}.$$

Across all participants, response times were slower for negatively skewed gambles compared to symmetric gambles (see Fig. S7, Table S5). As is typical, there was a main effect of age on response time, where older participants were slower to respond than younger participants. However, this effect was conditioned by an interaction with gamble acceptance: while older adults were slower to *accept* gambles than reject them, younger adults were slower to *reject* gambles than accept them. This interaction was further conditioned by gamble type: older adults were faster at accepting positively skewed gambles than symmetric gambles.

| **Table S5**  Linear regression models predicting response time | | |
| --- | --- | --- |
| Variables | Comparison | Behavior |
| Intercept |  | 1.44 [1.35, 1.54] |
| Age |  | **0.13 [0.03, 0.23]** |
| Gamble |  | 0.01 [−0.05, 0.06] |
| Skewness | Positive > Symmetric | 0.02 [−0.04, 0.07] |
|  | Negative > Symmetric | **0.09 [0.04, 0.14]** |
| Age by Gamble |  | **0.08 [0.02, 0.14]** |
| Skewness by Age | Positive > Symmetric × Age | 0 [−0.06, 0.05] |
|  | Negative > Symmetric × Age | 0.03 [−0.03, 0.08] |
| Skewness by Gamble | Positive > Symmetric × Gamble | −0.05 [−0.12, 0.03] |
|  | Negative > Symmetric × Gamble | −0.05 [−0.13, 0.02] |
| Skewness by Age by Gamble | Positive > Symmetric × Age × Gamble | **−0.08 [−0.16, 0]** |
|  | Negative > Symmetric × Age × Gamble | −0.02 [−0.09, 0.06] |
| AIC |  | 4,368.73 |
| BIC |  | 4,460.39 |
| Pseudo *R*^2^ |  | .21 |
| *Note*. Unstandardized betas (and 95% confidence interval) reported. Participants modeled as random effects. Significant fits highlighted in bold | | |

**Fig. S7** Response times over Age × Gamble Type. Left panel shows trials where the participants rejected the gamble, right panel displays trials where participants accepted the gamble

References

Knutson, B., & Greer, S. M. (2008). Anticipatory affect: neural correlates and consequences for choice. *Philosophical Transactions of the Royal Society B: Biological Sciences*, *363*(1511), 3771–3786. doi:10.1098/rstb.2008.0155

Leong, J. K., Pestilli, F., Wu, C. C., Samanez-Larkin, G. R., & Knutson, B. (2016). White-matter tract connecting anterior insula to nucleus accumbens correlates with reduced preference for positively skewed gambles. *Neuron*, *89*(1), 63–69.

Samanez-Larkin, G. R., Gibbs, S. E. B., Khanna, K., Nielsen, L., Carstensen, L. L., & Knutson, B. (2007). Anticipation of monetary gain but not loss in healthy older adults. *Nature Neuroscience*, *10*, 787–91. doi:10.1038/nn1894

Samanez-Larkin, G. R., Worthy, D. A., Mata, R., McClure, S. M., & Knutson, B. (2014). Adult age differences in frontostriatal representation of prediction error but not reward outcome. *Cognitive, Affective & Behavioral Neuroscience*, *14*, 672–82. doi:10.3758/s13415-014-0297-4
